# Identification of novel mutations by targeted exome sequencing and the genotype-phenotype assessment of patients with achromatopsia

**DOI:** 10.1186/s12967-015-0694-7

**Published:** 2015-10-22

**Authors:** Fen-Fen Li, Xiu-Feng Huang, Jie Chen, Xu-Dong Yu, Mei-Qin Zheng, Fan Lu, Zi-Bing Jin, De-Kang Gan

**Affiliations:** The Eye Hospital of Wenzhou Medical University, The State Key Laboratory Cultivation Base and Key Laboratory of Vision Science, Ministry of Health, Wenzhou, 325027 China; Department of Ophthalmology, Eye and ENT Hospital of Fudan University, 83 Fen Yang Road, Shanghai, 200031 People’s Republic of China

**Keywords:** Targeted exome sequencing, Genotype-phenotype, Novel mutations, Achromatopsia, Genetic diagnosis

## Abstract

**Background:**

Achromatopsia (ACHM) is a severe congenital autosomal recessive retinal disorder caused by loss of cone photoreceptors. Here, we aimed to determine the underlying genetic lesions and phenotypic correlations in two Chinese families with ACHM.

**Methods:**

Medical history and clinical evaluation were obtained from both families. Targeted exome sequencing (TES) was performed on 201 disease-causing genes of inherited retinal dystrophies to screen for ACHM causative mutations in the two probands.

**Results:**

The compound heterozygous mutations in *CNGA3* (c.1074G > A, p.W358X; c.1706G > A, p.R569H) were identified in the first proband, and a novel homozygous mutation (c.968C > A, p.A323D) was detected in the other pedigree. The proposed topological model of the *CNGA3* polypeptide suggested that the missense mutations primarily affected the transmembrane helix 5 and the cGMP-binding domain, respectively. Crystal structure modeling of the cyclic nucleotide-gated cation channel α-3 (CNGA3) protein encoded by the *CNGA3* gene revealed an abnormal combined structure generated by R569H.

**Conclusions:**

We firstly used the TES approach to identify genetic alterations in patients with ACHM. We uncovered three mutations in *CNGA3*, including one novel mutation. Our results not only expand the genotypic spectrum for *CNGA3* mutations, but also demonstrate that the TES approach is a valuable tool for molecular diagnosis.

**Electronic supplementary material:**

The online version of this article (doi:10.1186/s12967-015-0694-7) contains supplementary material, which is available to authorized users.

## Background

Achromatopsia (ACHM) is an early-onset and mostly stationary retinal dystrophy characterized by amblyopia (severely reduced visual acuity), pendular nystagmus, photophobia, and reduced or complete loss of color discrimination [1]. Currently, the diagnosis of ACHM is based on medical history, typical clinical manifestations, color discrimination problems in color vision testing, absent or reduced photopic (cone) responses and normal scotopic (rod) responses in electroretinograms (ERGs), and the presence of a normal fundus or only minor changes upon fundus photography [2].

To date, five causative genes (*CNGA3*, *CNGB3, GNAT2, PDE6C* and *PDE6H*) have been identified in ACHM patients [1, 3–6]. These genes encode crucial components of the cone phototransduction cascade. Mutations in *CNGA3* and *CNGB3*, which encode the α-subunit and β-subunit of the CNG, account for 25 % and 40–50 % of affected individuals in multiple ethnic groups, respectively [3, 7]. Mutations in *CNGA3* are considered the most common cause of ACHM and cone-rod dystrophies (CORDs) in Chinese, in which only cone photoreceptors are usually affected, although *CNGA3* mutations have been reported in a patient with CORDs and Leber congenital amaurosis (LCA) [8, 9]. However, approximately 20–30 % of ACHM cases appear to lack pathogenic mutations, which is likely due to the limitations in the number of screened regions [1, 3–5, 10].

Traditional techniques using Sanger sequencing for molecular diagnosis have several limitations, including being time-intensive and inconvenient for large scale analysis. Next-generation sequencing (NGS) has been shown to identify variants rapidly and systematically on an extremely large scale, which has greatly accelerated the development of gene discovery and molecular diagnosis [11]. NGS is increasingly being used for discovery of causative genes of Mendelian diseases and for genetic diagnosis [12, 13]. Targeted exome sequencing (TES) is an efficient method of NGS based on custom designed capture panels. TES can be used to identify disease-causing genes and to screen for mutations in hundreds of loci in genetically heterogeneous diseases. TES is less costly than whole genome sequencing (WGS) for mapped chromosomal regions, as well as for whole exome sequencing (WES). Thus, TES has several advantages compared to other approaches for reducing costs while enriching for discovery of highly penetrant variants [14], and for identifying pathogenic mutations with respect to both efficiency and accuracy. To date, some studies performed WES and detected causative mutations of ACHM [15, 16], but these lacked the TES approach to aid in molecular diagnosis of ACHM. Considering the enormous genotypic and phenotypic heterogeneity of inherited retinal dystrophies, there remains great potential to discover novel mutations or genes. In our study, we performed TES to screen 201 disease-causing genes of inherited retinal dystrophies in two families with ACHM, and discovered novel disease causative mutations, which broaden the spectrum of ACHM in Chinese.

## Methods

### Participants and clinical evaluations

**Fig. 1:**
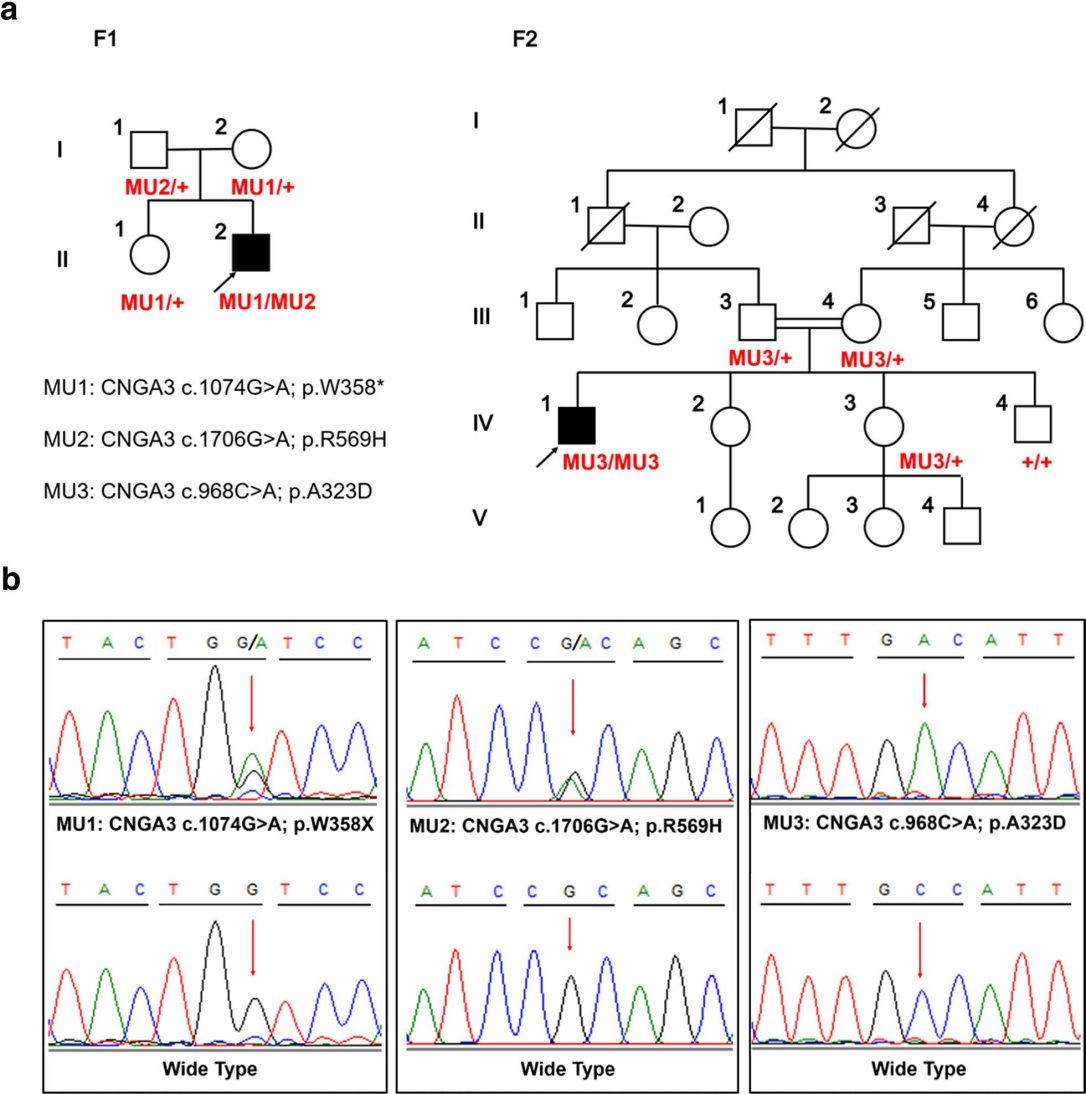
Pedigrees and identified mutations. **a** Pedigrees of two families were demonstrated with *CNGA3* genotypes was annotated to included family members. Probands are indicated by *arrows*. *Circles* females; *squares* males; *filled symbols* affected patients; *empty symbols* unaffected controls. **b** DNA sequencing profiles of the identified mutations (*upper*) and their wild-type form (*under*)

The study protocol was designed in adherence to the tenets of the Declaration of Helsinki, and was approved by the Ethics Committee of the Eye Hospital of Wenzhou Medical University. Nine participants from two unrelated families, including two patients and seven unaffected family members (Fig. 1a) were recruited to this study. Informed consent was obtained from all participants or their statutory guardian prior to the study. Patients’ initial symptoms and complaints included poor visual acuity and pendular nystagmus at an early age. Patients received a clinical diagnosis based on medical history, routine ophthalmological examination, and specialized visual function testing, including color vision testing, fundus photography and ERGs. A detailed family history was obtained from the patients and/or their relatives, and peripheral blood samples were collected.

### Targeted exome sequencing

We extracted a minimum of 3 μg of genomic DNA from each blood sample and fragmented it in order to construct the DNA library. One patient from each family was selected for TES. Biotinylated single-strand DNA capture probes were hybridized in solution with the target library. Streptavidin-coated magnetic beads were used to wash, elute, and amplify the DNA, which was then subjected to NGS using an Illumina Solexa HiSeq 2000 sequencer. A previously described targeted capture panel including 201 disease-causing genes (Additional file 1: Table S1) of inherited retinal dystrophies was applied for mutation screening in both pairs of patients [17].

### In-depth bioinformatics analyses and Sanger sequencing

The following databases were used for annotation of all identified variants: HapMap Project (http://hapmap.ncbi.nlm.nih.gov/), dbSNP137 (http://hgdownload.cse.ucsc.edu/goldenPath/hg19/database), 1000 Genome Project (ftp://1000genomes.ebi.ac.uk/vol1/ftp), Exome Variant Server (http://evs.gs.washington.edu/EVS/) and ExAC (http://exac.broadinstitute.org/). For variants that passed the initial filtration, we performed Sanger sequencing (Fig. 1b) and segregation analysis of the candidate mutant alleles in each patient’s family. To evaluate the different identified sequence variations, we analyzed 200 healthy control DNA samples.

### In silico analyses

The pathogenicity of the variant was assessed using in silico predicting online available programs, including PolyPhen-2 (http://genetics.bwh.harvard.edu/pph2/), SIFT (http://sift.jcvi.org/www/SIFT_enst_submit.html), and MutationTaster (http://www.mutationtaster.org/) for missense variants. Together, these assessments provided information on the pathogenicity of the variant. The topological model of the CNGA3 polypeptide was predicted using SMART (http://smart.embl-heidelberg.de/). The crystal structures of the wild-type and mutant proteins were predicted using Phyre2 (http://www.sbg.bio.ic.ac.uk/phyre2/html/page.cgi?id=index) [18] and visualized with PyMol software (Version 1.5). The model structure of the human cone CNG comprised of two CNGA3 and two CNGB3 subunits was previously described [19].

## Results

### Clinical findings

One patient from each family was clinically diagnosed with ACHM, and their detailed clinical features and mutations were summarized in Table 1. F1-II:2 reported to have evident nystagmus within the first few months after birth. Coincidentally, F2-IV:1 is a 29 year-old man with evident poor vision accompanied by nystagmus. The fundus presented with temporal disc pallor in F2-IV:1, and F1-II:2 only showed poor foveal reflex. There was no family history of ophthalmic disease, but the patient F2-IV:1 was from a consanguineous family. Both patients had normal anterior segments, ocular media, severe photophobia, and were unable to perform color-discrimination tasks. ERGs revealed severely reduced cone responses and normal rod response. Since ERGs were incomplete in young children, we only obtained results from F2-IV:1. All clinical manifestations were typical of complete ACHM.

**Table 1:** Clinical features of the ACHM patients in this study

Patient ID	Gene	Mutation	Gender	Age		Clinical manifestation			BCVA		Fundus appearance		ERGs	
				Exam	Onset	NYS	PP	PV	OD	OS	OD	OS	Rods	Cones
Family1-II:2	*CNGA3*	c.1074G > A; c.1706G > A	M	6 years	2 months	+	+	+	0.05	0.05	PFR	PFR	NA	NA
Family2-IV:1	*CNGA3*	c.968C > A; c.968C > A	M	29 years	<7 years	+	+	+	0.12	0.12	TDP	TDP	Normal	Severely reduced

*M* male, *NYS* nystagmus, *PP* photophobia, *BCVA* best corrected visual acuity, *OD* right eye, *OS* left eye, *PV* poor vision, *PFR* poor foveal reflex, *TDP* temporal disc pallor, *ERGs* electroretinograms, *NA* not available

### Mutations identified by TES

We used a panel covering 201 disease-causing genes of inherited retinal dystrophies to screen for ACHM mutations. Our TES reached an average mean depth of 168X with greater than 97.8 % coverage of the targeted regions. After alignment and bioinformatics analyses, single nucleotide variants (SNVs) and insertions/deletions (Indels) were annotated to the exome databases, of which those with MAF >0.005 or found homozygous >1 subject were filtered. The missense variants were discarded with tolerant prediction using in silico tools. Candidate variants were then confirmed in the family members. In total, compound heterozygous mutations and one homozygous mutation in the *CNGA3* gene were identified in the two families (Fig. 1a, b). In family 1, the proband F1-II:2 harbored two different heterozygous mutations in CNGA3 (c.1074G > A, p.W358X; c.1706G > A, p.R569H), which were demonstrated to originate from the paternal and maternal allele, respectively. His healthy sister carried a heterozygous mutation (c.1074G > A, p.W358X). Both of these mutations were identified in previous studies [7, 8, 20]. In family 2, the proband F2-IV:1 harbored one homozygous mutation in CNGA3 (c.968C > A, p.A323D), as a result of being consanguineous to the F2 family. His healthy sister (IV:3) carried a heterozygous mutation and brother (IV:4) has normal genotype. To the best of our knowledge, this is the first report of the A323D mutation. These two missense mutations were predicted to be deleterious based on three types of online predictive software (Table 2). We aimed to detect whether any of the three potential mutations existed in normal populations, so direct Sanger sequencing of the three points were applied in 200 healthy controls. Finally, all the three potential mutations were absent in the 200 controls.

**Table 2:** *CNGA3* mutations identified in the study

Family ID	Exon	Variation				PolyPhen2	SIFT	Mutation taster	DbSNP	References
		Nucleotide	Protein	Status	Type					
Family1	7	c.1074G > A	p.W358X	Het	Nonsense	–	–	–	Novel	[8]
Family1	7	c.1706G > A	p.R569H	Het	Missense	PD (0.991)	D (0.00)	DC (0.999)	Novel	[7, 20]
Family2	7	c.968C > A	p.A323D	Hom	Missense	PD (0.960)	D (0.02)	DC (0.999)	Novel	This study

*Het* heterozygous, *Hom* homozygous, *D* deleterious, *PD* probably damaging, *DC* disease-causing

### Structural modeling

**Fig. 2:**
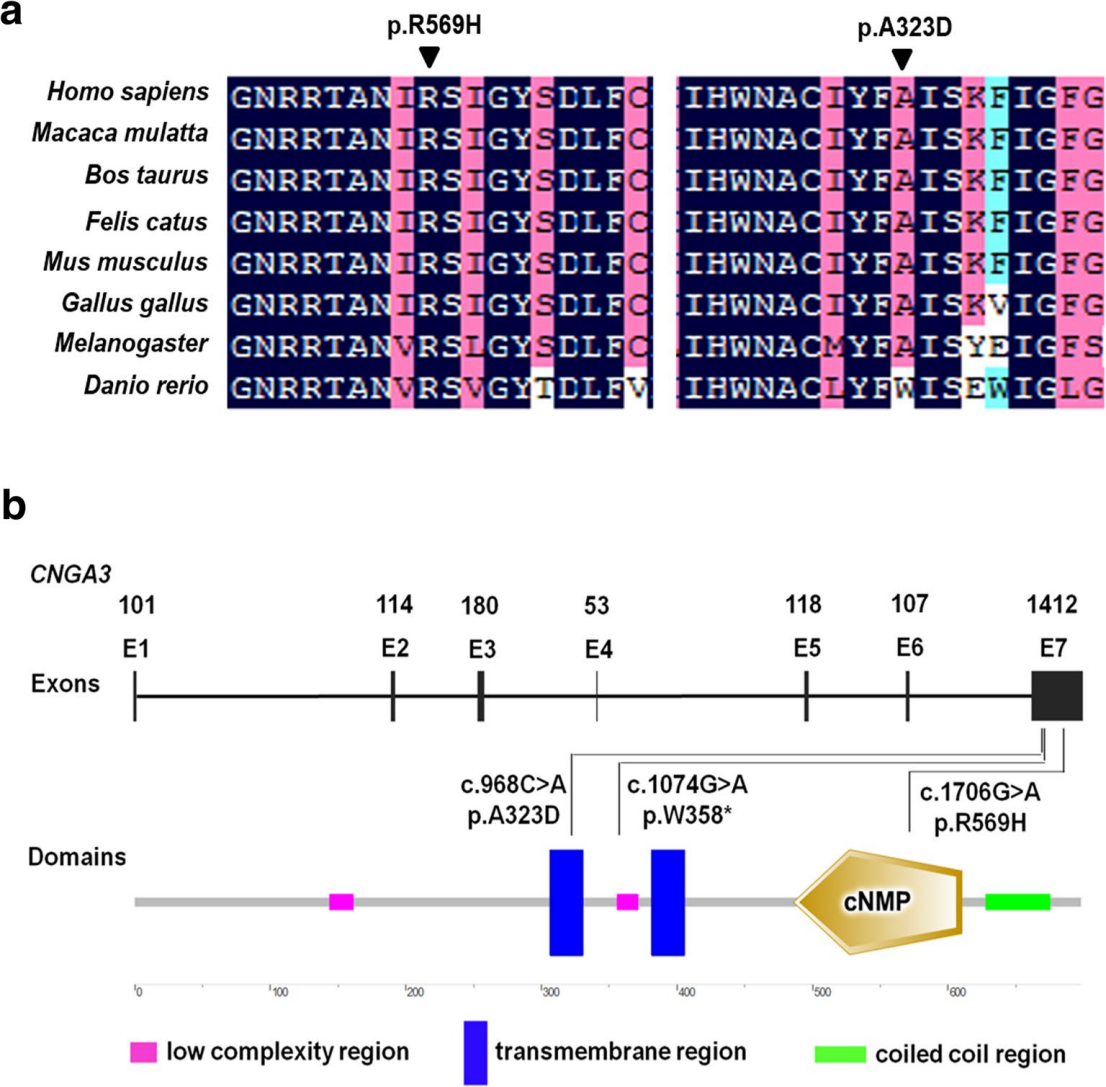
Mutations identified in two families. **a** Multiple sequence alignment of *CNGA3* polypeptides of different species showing the conserved amino acid residues (Arginine 569 and Alanine 323). **b** Exons of the *CNGA3* gene (*upper*) and location of the mutations with respect to the topological model of the CNGA3 polypeptide

**Fig. 3:**
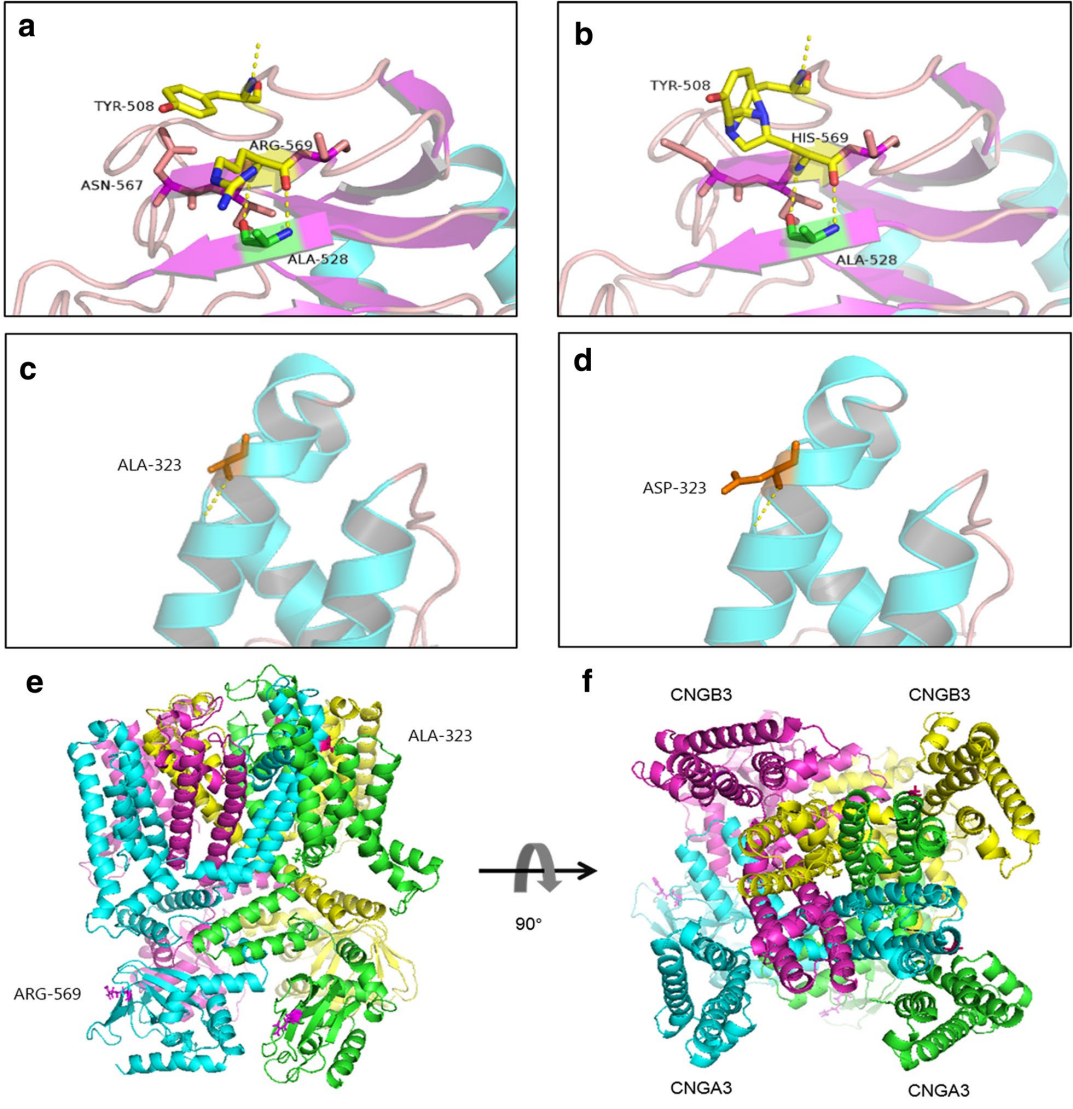
Predicted crystal structures of CNGA3 protein. Predicted crystal structures of wild-type (**a**, **c**) and mutant (**b**, **d**) CNGA3 protein. Side view (**e**) and extracellular view (**f**) of the transmembrane domain of the cone CNG model structure. The proposed locations of the two CNGA3 and two CNGB3 subunits are marked. *Magenta* coloring indicates residue 569, while *pink* represents residue 323

The first G-A substitution (W358X) in exon 7 of the *CNGA3* gene is predicted to generate a premature termination codon (PTC) at residue 358 of the CNGA3 protein (Fig. 2a). The second mutation (R569H) is a missense mutation that changes Arginine to Histidine at residue 569. The A323D mutation was a novel missense substitution from Alanine to the Aspartic acid at residue 323. The proposed topological model of the *CNGA3* polypeptide included the six transmembrane helices (S1–S6), the ion pore, and the cGMP-binding domain [7, 21]. Both the R569H and A323D missense mutations affected evolutionarily highly conserved amino acid residues in *CNGA3*; R569H is located in the cGMP-binding domain while A323D mostly affects transmembrane helix S5 (Fig. 2a, b). Structural modeling revealed the generation of a novel bond between the mutated Histidine at residue 569 and Tyrosine at residue 508 (Fig. 3a, b). The other homozygous mutation (A323D) changes Alanine to Aspartic acid (Fig. 3c, d). The model structure of the human cone CNG, which is comprised of two CNGA3 (α) and two CNGB3 (β) subunits (Fig. 3e, f), revealed that both mutations affect highly conserved residues. Although there was no apparent change in the œ-subunits. These mutations significantly affect the combination between α-subunit and ß-subunit. Structural modeling was constructed on the basis of the crystal structure of template c4chwB [18], which demonstrated sequence coverage of 49 % with the protein cyclic nucleotide-gated ion channel α-subunit.

## Discussion

Achromatopsia (ACHM) is a severe congenital autosomal recessive retinal disorder caused by loss of cone photoreceptors. Mutations in the *CNGA3* gene are recognized as the most common causes of ACHM and cone-rod dystrophies in the Chinese population [8]. In our study, we report three distinct disease-causative *CNGA3* mutations, including one novel variant and two known mutations, in two Chinese families with ACHM. Two heterozygous mutations in F1 have been previously reported, however this type of compound *CNGA3* mutation has not been described to date. Furthermore, we reveal for the first time a novel homozygous missense (A323D) in *CNGA3* that leads to ACHM.

The *CNGA3* gene, located on chromosome 2q11, contains 7 exons. *CNGA3* and *CNGB3* encode the α- and β-subunits that composed to the cone CNG. Heterologous expression studies have shown that the α-subunits are responsible for the ion-conducting activity of the channel, whereas the β-subunits function as modulators [21]. A study using a homologous *Cnga3* knockout-mouse model demonstrated complete absence of physiologically measurable cone function, a decrease in the number of cones in the retina, and morphological abnormalities of the remaining cones [22]. The compound heterozygous mutations, W358X and R569H, are unable to retain crucial functional activity. The R569H heterozygous and A323D homozygous mutations are mainly confined to the functionally and structurally important central parts of the *CNGA3* polypeptide, affecting the S5 and the cGMP-binding domains, respectively. Crystal structure modeling suggests that R569H impacts on the generation of bonds, while A323D is not predicted to significantly influence the spatial conformation of the protein. However, the addition of hydrogen could potentially affect the association with the β-subunit, as both mutants localize to highly conversed residues.

Patients in both families showed typical clinical manifestations of ACHM. The importance of *CNGA3* mutations is underscored by the finding that these mutations not only cause complete ACHM, but also incomplete ACHM [7]. With respect to incomplete ACHM, combined clinical diagnosis with diagnostic panels used for genetic testing can improve the sensitivity of diagnosis. Ophthalmological evaluation, including color vision testing, fundus photography and ERGs. Color vision testing was unable to be performed in cases of severe photophobia and poor visual acuity. Fundus photography and ERGs are time-consuming, and can be stressful for the patient. Moreover, clinical investigations are sometimes incomplete in children or might require general anaesthesia. Moreover, genetic testing in the patients allowed a focused analysis opportunity for genetic risk assessment and genetic counselling [2].

To date, a small number of studies have focused on gene therapy in patients carrying mutations in the *CNGA3* gene [23, 24]. Using the novel TES approach described in our study, one can confirm the clinical diagnosis and rapidly identify the mutations, which greatly enables therapeutic intervention and personalized medicine.

## Conclusions

In summary, we used the paneled exome sequencing methodology for molecular diagnosis of inherited ACHM, and identified three mutations in two patients, including two compound mutations and one novel homozygous mutation. TES can be used for both investigative and diagnostic purposes as this technique can identify the disease causing mutations. Genetic testing is a useful tool to complement diagnostic procedures, and TES can be of particular benefit to children with early-onset ACHM.

## Additional file

10.1186/s12967-015-0694-7 The list of 201 disease-causing genes of capture panel.

## Abbreviations

ACHM: achromatopsia
ERGs: electroretinograms
CORDs: cone-rod dystrophies
LCA: leber congenital amaurosis
NGS: next-generation sequencing
TES: targeted exome sequencing
WGS: whole genome sequencing
WES: whole exome sequencing
SNVs: single nucleotide variants
