# Supplementary material for: Identification of novel mutations by targeted exome sequencing and the genotype-phenotype assessment of patients with achromatopsia
Source: J Transl Med. 2015 Oct 22;13:334. doi: 10.1186/s12967-015-0694-7 (PMC4618873; doi:10.1186/s12967-015-0694-7)
Supplement: Supplementary file 1 — 10.1186/s12967-015-0694-7 The list of 201 disease-causing genes of capture panel. [file 12967_2015_694_MOESM1_ESM.docx]

**Table S1. The list of 201 disease-causing genes of capture panel.**

| *ABCA4;ABCC6;ABHD12;ACBD5;ADAM9;ADAMTS18;AHI1;AIPL1;ALMS1;ARL2BP;ARL6;ATXN7;BBIP1;BBS1;BBS10;BBS12;BBS2;BBS4;BBS5;BBS7;BBS9;BEST1;C1QTNF5;C2orf71;C8orf37;CA4;CABP4;CACNA1F;CACNA2D4;CAPN5;CC2D2A;CDH23;CDH3;CDHR1;CEP164;CEP290;CERKL;CFH;CHM;CIB2;CLN3;CLRN1;CNGA1;CNGA3;CNGB1;CNGB3;CNNM4;COL11A1;COL2A1;COL9A1;CRB1;CRX;CYP4V2;DFNB31;DHDDS;DMD;DTHD1;EFEMP1;ELOVL4;EMC1;EYS;FAM161A;FLVCR1;FSCN2;FZD4;GNAT1;GNAT2;GNPTG;GPR125;GPR179;GPR98;GRK1;GRM6;GUCA1A;GUCA1B;GUCY2D;HARS;HMCN1;IDH3B;IFT140;IMPDH1;IMPG1;IMPG2;INPP5E;INVS;IQCB1;ITM2B;JAG1;KCNJ13;KCNV2;KIAA1549;KIF11;KLHL7;LCA5;LRAT;LRIT3;LRP5;LZTFL1;MAK;MERTK;MFN2;MFRP;MKKS;MKS1;MTTP;MVK;MYO7A;NDP;NEK2;NMNAT1;NPHP1;NPHP3;NPHP4;NR2E3;NRL;NYX;OAT;OFD1;OPA1;OPA3;OPN1LW;OPN1MW;OPN1SW;OTX2;PANK2;PAX2;PCDH15;PDE6A;PDE6B;PDE6C;PDE6G;PDE6H;PDZD7;PEX1;PEX7;PGK1;PHYH;;PITPNM3;PLA2G5;PRCD;PROM1;PRPF3;PRPF31;PRPF6;PRPF8;PRPH2;PXMP3;RAB28;RAX2;RB1;RBP3;RBP4;RD3;RDH12;RDH5;RGR;RGS9;RGS9BP;RHO;RIMS1;RLBP1;ROM1;RP1;RP1L1;RP2;RP9;RPE65;RPGR;RPGRIP1;RPGRIP1L;RS1;SAG;SDCCAG8;SEMA4A;SNRNP200;SPATA7;TEAD1;TIMM8A;TIMP3;TMEM237;TOPORS;TREX1;TRIM32;TRPM1;TSPAN12;TTC8;TTPA;TULP1;UNC119;USH1C;USH1G;USH2A;VCAN;WDPCP;WDR19;WFS1;ZNF423;ZNF513;SLC7A14;SLC24A1;SNRNP200;SPATA7;TIMM8A;TIMP3;TMEM126A;TOPORS;TRIM32;TRPM1;TSPAN12;TTC8;TTPA;TULP1;UNC119;USH1C;USH1G;USH2A;VCAN;WFS1;ZNF513* |
| --- |
